# Opening a Window to the Autistic Brain

**DOI:** 10.1371/journal.pbio.0020267

**Published:** 2004-08-17

**Authors:** Kendall Powell

## Abstract

Research focuses have shifted from "curing" autism to finding better diagnostics for early intervention, improving behavioral therapies, and gaining insight into the autistic brain

At first glance, the preschool classroom on the other side of the two-way mirror looks like any other—brightly colored rugs, scattered toys, and tiny chairs. But almost immediately an observer notices differences in the Team Toddle students here at the Neuropsychiatric Institute of the University of California at Los Angeles (UCLA) (Los Angeles, California, United States).

A therapist instructs a toddler on his colors, flashing a rapid sequence of blocks at him. When the toddler starts rocking in his chair and repeatedly touching his forehead, the therapist physically restrains his hands, placing them back on the tabletop until he stops the repetitive behaviors and focuses once again on her face and the blocks. During playtime, a two-year-old girl sits by herself in the corner, fixated on some picture cards, oblivious to a group of other children playing with a racetrack and to the therapist who tries to draw her out to join the group.

These children lack some of the key social skills that normal toddlers pick up naturally—looking to others for reassurance or cues, focusing on faces, and playing together. Social and communication impairment is a hallmark of autism and can show up as early as 12–18 months of age. But with an unknown cause, and genetic linkages still hazy, there is little consensus among researchers on how the disorder develops in children and how it causes a broad spectrum of social, language, and behavioral deficits.

Following one line of research, David Amaral's laboratory at the M.I.N.D. Institute at the University of California at Davis Medical Center in Sacramento (California, United States) has recorded, in autistic brains, a brain volume increase in a specific structure, the amygdala, which is thought to be important for social behavior. A similar study at the University of Washington in Seattle (UW) (Seattle, Washington, United States) has reached the same conclusion. “There are so few facts about autism, to have two labs come up with the same data is phenomenal,” says Amaral. “We feel confident this is a real finding, but what does it mean to these kids?”

On another research track, using functional imaging, Ralph-Axel Müller, a cognitive neuroscientist at San Diego State University (San Diego, California, United States) sees a scattering of brain activation in autistic brains that he views as an indication of a more general brain development problem underlying the disorder (Figure 1). He has hypothesized that the early-developing basic functions may require more brain area in autism, pushing out and disturbing the later specialization for more complex functions. “I'm sure this is wrong,” he says, “but it will allow us to look in a more hypothesis-driven way at animal studies of how the cerebral cortex develops specialization.” Animal models may, in turn, yield clues about normal and abnormal brain development in humans.

**Figure 1:**
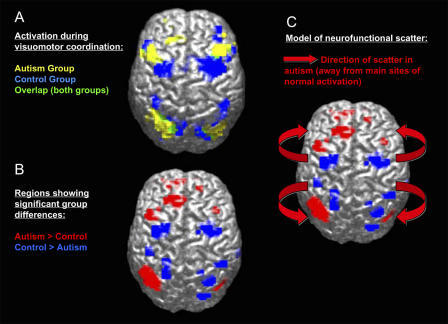
Brain Activation Scattering in Autism Autistic individuals show less activity, during a movement task, in areas that are normally activated (premotor and superior parietal cortex; blue areas), but unusually increased activity around these normal sites of activation (red areas). Images courtesy of Ralph-Axel Müller.

“Since there is no major hypothesis as to cause [of autism], there are many plausible ideas,” says Amaral. “If we go after all of them, we will waste all of our resources. [We have to] come to some consensus about which are most plausible.” At least two levels of pursuit exist for tracing brain problems associated with autism—the exploration of the general developmental disruptions that result in an autistic brain, and the examination of more specific problems in particular brain structures that produce symptoms. Although scientists still debate how autism evolves in a patient, the field has begun in the last decade to replicate findings and make science-based arguments for interventions. Progress has come in small steps, with advances in neuroimaging and more rigorous experimental designs.

Research focuses have shifted from “curing” autism to finding better diagnostics for early intervention, improving behavioral therapies, and gaining insight into the development and function of the autistic brain. Both advocacy groups and government programs have started to bring together neuroscience and genetics experts, clinicians, and families to sharpen the focus of studies and ensure progress in what has often been a messy field.

## A World Apart

Autism spectrum disorder strikes between one and six out of every 1,000 children around the world, but diagnosis and treatment are currently limited to developed countries. Autism is four times more prevalent in boys than girls, but makes no racial, ethnic, or socioeconomic distinctions. It is characterized by three main symptoms: impaired language, social and communicative deficits, and repetitive and stereotyped behaviors, such as hand flapping, rocking, and unusual responses to sensory stimuli. Autism spectrum disorders can be broken down into other categories, such as low-functioning autism (IQ below 70), high-functioning autism (IQ above 70), and Asperger syndrome (similar to high-functioning autism but with no language deficit).

Researchers suspect that there are even more distinct subsets of autism patients. For example, some patients also have epilepsy, and it has been suggested that there is a regressive form of autism—children who, at two or three years of age, appear to regress and lose developmental milestones they had already achieved. Researchers say that sorting out these different profiles—or phenotypes—of autism will be especially important in sorting out which genes or which brain abnormalities are implicated for particular deficits. This sorting should also help clarify the mounds of contradictory data that have dogged the field, by tamping down the experimental “noise” in studies. Boosting the number of children studied and following them from early infancy through adolescence and beyond will also be key components of future studies.

“There is not going to be rapid progress in autism research unless we subtype,” Amaral says. He predicts that “brain differences in kids with a regressive form of autism will be different than those of kids with the more congenital type of autism.” He and others are teaming up in an autism phenotyping project that will characterize 600 children into categories of autism (comparing them to 600 children with mental retardation and 600 controls). Splitting autism into subtypes will boost both neurobiology and genetics studies (Box 1) to find real effects related to specific traits.

## Facing Up to Autism

A key area of research explores the brain's response to human faces at a young age. Studies at the UW Autism Center have shown that unlike typically developing three-year-olds, autistic children do not show a differential brain response to their mother's face compared to that of a stranger. While dysfunctional face recognition may be one of the more devastating symptoms for caregivers, it is also one of the most promising avenues for research to determine how autistic brains process their world differently.

Sara Webb, a child psychologist at UW, has followed about 70 autistic children since the age of three for a longitudinal study that will test many parameters until they reach age nine. Her work has already shown that autistic three-year-olds process seeing a strange toy differently from seeing a favorite toy, in the same way a normal child does. But activity in their brains—measured through a network of electrodes placed on the scalp—is similar whether the face is familiar (for example, mom) or strange. This, Webb says, led to two hypotheses: either the brain area for face processing is not set up correctly in autistic children, or the way these children incorporate experiences from their environment is so different that the brain area develops improperly.

“We think the latter is a more likely explanation at this point,” says Webb. “By the time they are adolescents or adults, they are showing the [proper] response for familiar faces.” Indeed, a functional MRI (fMRI) study by UW neuroimaging researcher Elizabeth Aylward showed that the brains of high-functioning adolescents and adults did activate the face-recognition center, the fusiform gyrus, when shown a very familiar face. However, the same subjects did not activate the center when viewing strange faces. This points to the possibility that greater experience seeing the familiar face (i.e., on a daily basis for many years) can eventually influence the appropriate brain areas.

“You need the biological wiring set up properly, but you also need experience for it to function normally,” says Aylward. “We're guessing what is missing is the experience.” To test that idea, one of her graduate students will “train” half of the autistic patients in face recognition—something most children pick up on their own—by having them study, manipulate, and match faces using computer games. Then fMRI scans will be done again to see if the fusiform gyrus might now be activated when viewing strange faces, as it is in control subjects. Intense training of a similar type for reading has already been shown to effect change in brain activation in as little as three weeks for children with dyslexia.

In their model, it is as if “all the parts are there, ready to go, but somehow they haven't gotten the ignition turned on,” says Aylward. At the 2004 annual meeting of the American Association for the Advancement of Science (Washington DC, United States), the UW center director Geraldine Dawson explained that this tackling of specific deficits will help researchers attach them to particular “mind modules” in the brain and will ultimately lead to the genes that control the development or function of those modules. That modular view, however, is not shared by many of her colleagues elsewhere, who argue that autistic behaviors are the result of a system-wide perturbation of early brain development and connectivity.

## Structural Support

For example, Müller points to structural studies that seem to uphold his theory of overall disorganization of the brain's cortex. Work by Manuel Casanova and colleagues at the University of Louisville (Louisville, Kentucky, United States) shows that the “minicolumns” of neurons that make up the cortex are narrower and more numerous in autistic brains. Normally, these organized bundles appear very early in the developing fetal brain. In postmortem studies of autistic brains, Casanova found that the minicolumns had the same number of neurons, but smaller margins between the bundles. The margins, Casanova says, may act like “a shower curtain of inhibition that prevents information from flooding adjacent minicolumns.”

Reducing those margins, he hypothesizes, could mean that an autistic brain has too much positive feedback, acting like a noisy amplifier. “For an autistic individual who is trying to piece together too much information from a face, maybe it's like looking at the sun,” he says.

More general studies of adult autistic neuroanatomy have given conflicting results—most likely from diversity in the study populations—that make functional inferences difficult, if not impossible. But recent studies that focus on developing autistic brains earlier in life have revealed intriguing differences from normally developing children.

Several studies have shown that from ages two to four, autistic children have larger overall brain volumes (and correspondingly larger head circumferences) than normal children, but that the difference had disappeared by about age six or seven. Since autism is usually diagnosed around age two or three, when the brain is already abnormally large, Eric Courchesne and colleagues at University of California, San Diego (San Diego, California, United States) hypothesized that brain overgrowth must occur earlier, before signs of autism appear.

In an elegant retrospective study, the team analyzed head circumference and brain volume measurements of autistic children that started at birth and continued until 14 months of age. The study revealed that at birth, autistic children's head size is much smaller than healthy children, in the 25th percentile, but by 6–14 months, their head size had increased to the 84th percentile, an excessive growth rate. The increase correlated with increased brain volumes of both gray and white matter regions measured by structural imaging between ages two to five.

The Courchesne study strongly suggests that with autism, significant unregulated brain growth occurs in the first year of life. The team also found an association between greater increases in brain size in infancy and a later age for first word, worse repetitive behavior, and a trend toward more severe autistic symptoms later, at diagnosis. The rapid growth of autistic brains may produce too many connections too quickly, without the opportunity to be shaped by the experience and input that a typically developing child accumulates over many years. At age six or later, when the growth slows, the already derailed connections may no longer be able to incorporate experiences. “By that time,” write Courchesne et al., “the period of plasticity that allows the exquisite and graceful complexity of the human brain to emerge will have passed.”

## Playing Well with Others

This idea that autistic brains are developing at warp speed, to their detriment, fits intriguingly well with what is known about treatment of autism—the earlier and more intense behavioral therapy an autistic child receives, the better the outcome will be. That's why the toddlers at UCLA get one-on-one training by therapists, who fire rapid questions and physically repeat tasks until they sink in.

Stephanny Freeman, co-director of the Early Childhood Partial Hospitalization program at UCLA (Los Angeles, California, United States), says these methods would be alien to, and lost on, typically developing two-year-olds, who would be bewildered by such a highly structured environment. Her colleague and co-director, Tanya Paparella, chimes in, “It as if we are opening a window or door to the autistic brain.” Keeping that door open as long as possible in very young autistic patients seems to give them a better prognosis than older children, who are more difficult to treat.

**Figure 2:**
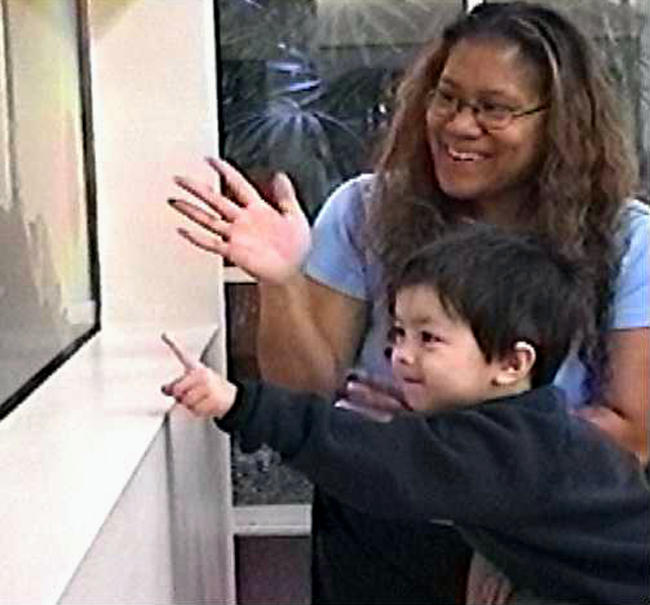
Pointing as an Example of Joint Attention A child with autism (three years old) pointing to the fish in an aquarium. Photo courtesy of Connie Kasari.

But while most agree that early and intense therapy is good for autistic children, until recently, little research on intervention methods existed. Connie Kasari, an educational psychologist at UCLA, along with Freeman and Paparella, has run one of the first randomized, controlled trials on therapies designed to teach autistic kids social skills. The group tested two skills in particular—sharing attention with others and pretend playing (Figure 2). The team hypothesizes that these skills, which normal children pick up easily and early, lay important groundwork for language development.

The team's results show that autistic children can learn these skills from intense training. At least anecdotally, some of these children have gone on to function in normal school classrooms, even making a few friends, although they are still a bit socially awkward. Whether or not improvements in those skills will correlate with language improvements will require further testing. But Kasari notes that this work is not universally accepted in the autism therapy community, and that many more controlled studies will have to be published before a system-wide change in autism preschool education can occur.

## Funding the Search

In the last decade, National Institutes of Health funding for autism research has increased from $10 million to $80 million, and much of that has been funneled into large, multidisciplinary research projects. Advocacy groups such as Cure Autism Now (Los Angeles, California, United States) and the National Alliance for Autism Research (Princeton, New Jersey, United States) greatly influence which autism research projects get funded, both through their own grant programs and also by lobbying Congress for increased federal grants. Some question whether it is wise to let emotions and the desire to find a cure drive research agendas. In the past, tensions between government programs and advocacy programs have run high.

Casanova, for one, criticizes the disproportionate flow of money to what he calls imaging and genetic “fishing expeditions” and says more should go to neuropathology studies. He points out that only about 40 postmortem, mostly adult, autistic brains have been studied so far, a tiny fraction compared to those studied in other neuropathological disorders like Alzheimer's disease or schizophrenia.

But Daniel Geschwind, a neurogeneticist at UCLA, defends this approach, saying that a well-planned fishing expedition that uses the right technology and looks in the appropriate places can result in a “freezer full of fish.” He also says that parent organizations keep the field honest by “constantly reminding us to keep an eye on the ball and don't get distracted.” Geschwind, Amaral, and other top experts have recently been recruited by advocacy groups or by friends with autistic children to shift some of their research questions to examining autism.

As more researchers in genetics and neuroscience have become involved, Amaral says, the tensions between the parent groups and the National Institutes of Health have eased. “The parents communicated to the scientists the tremendous need for research and the scientists convey back to them which [research projects] make sense to fund,” he says. He adds that advocacy groups have been indispensable to research, setting up large genetic and brain tissue banks and enlisting families to participate in those efforts.

So, researchers say, the goals of the National Institutes of Health programs and the advocacy programs have started to come together to focus on well-executed studies that might lead to better diagnostics and earlier, proven interventions. The work of Courchesne et al. suggests that children at risk for autism might easily be diagnosed by head circumference measurements as early as the first few months of life. Imaging studies combined with training programs, such as the work at UW on face recognition, may one day be able to verify that behavioral interventions are effective at activating target brain areas. As researchers work to untangle the causes and effects of brain dysfunctions in autism, Aylward notes, there is good reason to be hopeful: “Although this is a genetic disorder, we know there is plasticity in the young brain.”

**Box 1. Genetic Power-Up**

Evidence abounds that autism results from multiple gene mutations. Identical twins share an autism diagnosis 60%–95% of the time, and a younger sibling of an autistic child is 50 times more likely to have autism. There are also four times as many autistic males as females, indicating a possible sex chromosome difference in inheritance. Genetics researchers estimate that autism is the result of mutations in anywhere from 2 to 20 genes.

By studying the commonly inherited pieces of chromosomes in autistic siblings, geneticists have identified a handful of chromosome hotspots. However, each region contains hundreds of individual genes, and narrowing down to specific mutations will require studies that either involve thousands of families or tackle specific phenotypes. Daniel Geschwind, a neurogeneticist at UCLA, has already completed such a study. It reveals a linkage—the probability that a region contains a gene or genes linked to the disorder—between language deficits and a hotspot region on Chromosome 7. His team looked at a more homogenous group of autistic patients, all of whom had a similar language delay measured quantitatively by time to first spoken word.

“Endophenotypes measure something that underlies the disorder in a significant way and [therefore probably] also underlies a genetic component,” says Geschwind. “We're trying to identify characteristics that really underlie the genetic peaks of interest.” Another such study, by Margaret Pericak-Vance and colleagues at Duke University Medical Center (Durham, North Carolina, United States), used the characteristic of “insistence on sameness”—a subset of stereotyped behaviors such as resisting change in routine or environment, and compulsions. By running a genetic analysis on a group of patients with the highest “insistence on sameness” scores from diagnostic tests, the Duke team increased the linkage score and further narrowed the hotspot region on Chromosome 15.

## Abbreviations

fMRI: functional MRI
UCLA: University of California at Los Angeles
UW: University of Washington in Seattle

## References

[pbio-0020267-Alarcon1] Alarcón M, Cantor RM, Liu J, Gilliam C, Geschwind DH (2002). Evidence for a language quantitative trait locus on chromosome 7q in multiplex autism families. Am J Hum Genet.

[pbio-0020267-Amaral1] Amaral DG, Bauman MD, Schumann CM (2003). The amygdala and autism: Implications from non-human primate studies. Genes Brain Behav.

[pbio-0020267-Casanova1] Casanova MF, Buxhoeveden D, Gomez J (2003). Disruption in the inhibitory architecture of the cell minicolumn: Implications for autism. Neuroscientist.

[pbio-0020267-Courchesne1] Courchesne E, Carper R, Akshoomoff N (2003). Evidence of brain overgrowth in the first year of life in autism. JAMA.

[pbio-0020267-Dawson1] Dawson G, Carver L, Meltzoff AN, Panagiotides H, McPartland J (2002). Neural correlates of face and object recognition in young children with autism spectrum disorder, developmental delay, and typical development. Child Dev.

[pbio-0020267-Muller1] Müller R.-A, Kleinhans N, Kemmotsu N, Pierce K, Courchesne E (2003). Abnormal variability and distribution of functional maps in autism: An fMRI study of visuomotor learning. Am J Psychiatry.

[pbio-0020267-Shao1] Shao Y, Cuccaro ML, Hauser ER, Raiford KL, Menold MM (2003). Fine mapping of autistic disorder to chromosome 15q11-q13 by use of phenotypic subtypes. Am J Hum Genet.

